# A Temporal Signature of Epidermal Growth Factor Signaling Regulates the Differentiation of Germline Cells in Testes of *Drosophila melanogaster*

**DOI:** 10.1371/journal.pone.0070678

**Published:** 2013-08-05

**Authors:** Alicia G. Hudson, Benjamin B. Parrott, Yue Qian, Cordula Schulz

**Affiliations:** 1 Department of Cellular Biology, University of Georgia, Athens, Georgia, United States of America; 2 Department of Obstetrics and Gynecology, Medical University of South Carolina, Charleston, South Carolina, United States of America; University of Massachusetts Medical School, United States of America

## Abstract

Tissue replenishment from stem cells follows a precise cascade of events, during which stem cell daughters first proliferate by mitotic transit amplifying divisions and then enter terminal differentiation. Here we address how stem cell daughters are guided through the early steps of development. In *Drosophila* testes, somatic cyst cells enclose the proliferating and differentiating germline cells and the units of germline and surrounding cyst cells are commonly referred to as cysts. By characterizing flies with reduced or increased Epidermal Growth Factor (EGF) signaling we show that EGF triggers different responses in the cysts dependent on its dose. In addition to the previously reported requirement for EGF signaling in cyst formation, a low dose of EGF signaling is required for the progression of the germline cells through transit amplifying divisions, and a high dose of EGF signaling promotes terminal differentiation. Terminal differentiation was promoted in testes expressing a constitutively active EGF Receptor (EGFR) and in testes expressing both a secreted EGF and the EGFR in the cyst cells, but not in testes expressing either only EGF or only EGFR. We propose that as the cysts develop, a temporal signature of EGF signaling is created by the coordinated increase of both the production of active ligands by the germline cells and the amount of available receptor molecules on the cyst cells.

## Introduction

Tissue homeostasis depends on adult stem cells that constantly self-renew and produce differentiated cells [1], [2]. Self-renewal of stem cells and differentiation of stem cell daughters are regulated by interactions with other cell types. For example, in the hair follicle of the skin, melanocyte stem cells are closely associated with epithelial stem cells and signaling between the two lineages is an important mechanism in coordinating the differentiation of the two stem cell lineages to make pigmented hair [3], [4]. Also in the skin, follicular stem cell activation is regulated by signals from underlying intradermal adipocytes, and in the bone marrow, hematopoietic stem cell fate and proliferation depend on mesenchymal stem cells [5]–[7]. One of the best described examples of the dependence of a stem cell lineage on another cell type is the development of germline cells in the male gonad of *Drosophila melanogaster* [8].

**Figure 1:**
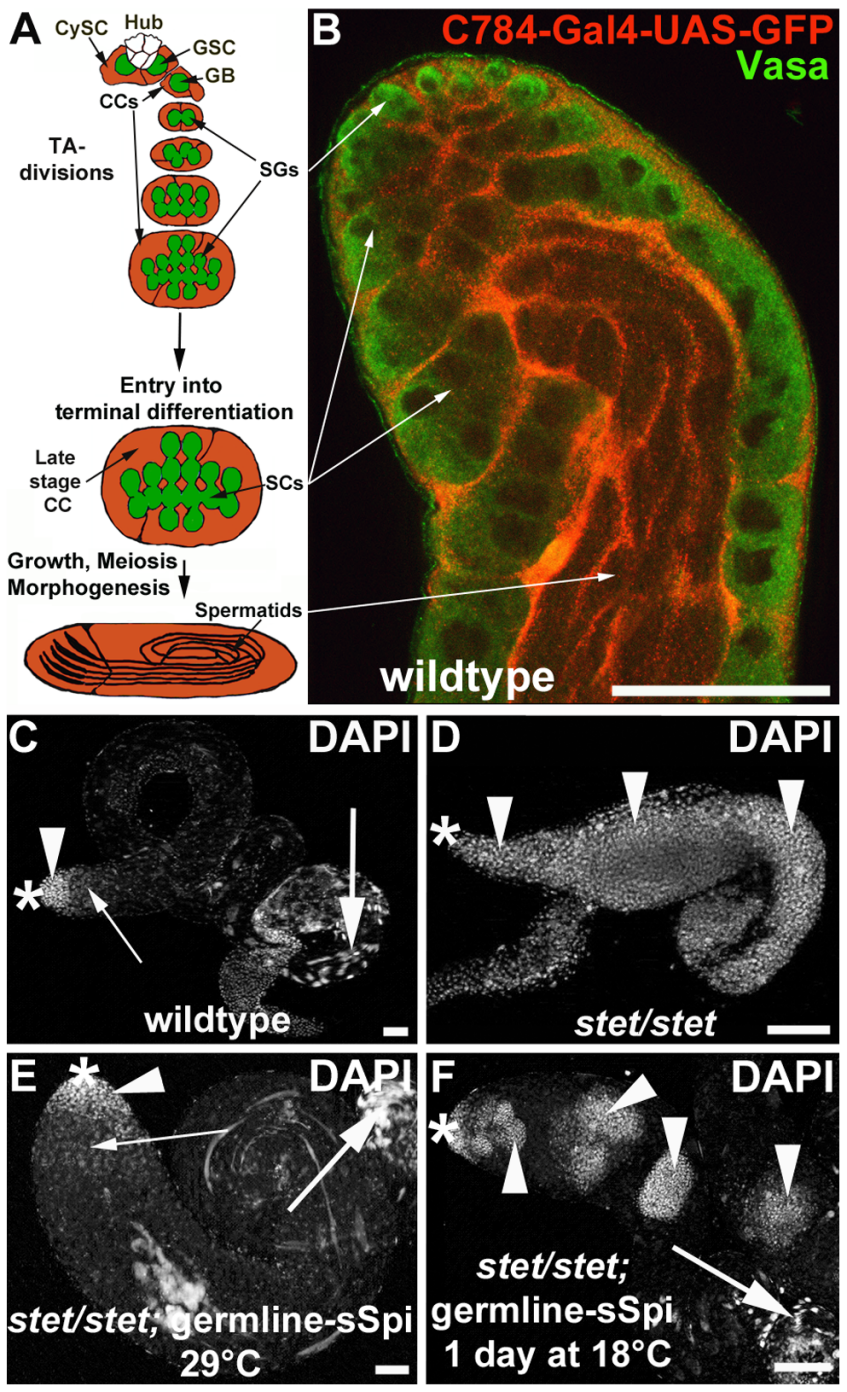
Reduction in EGF signaling disrupts germline differentiation. (A) Cartoon illustrating the cell types and their arrangement in a wildtype testis. Germline stem cells (GSCs) are located around the apical hub (white cells) and are enclosed by cyst stem cells (CySCs). Each gonialblast (GB) and its progeny are completely enclosed by two early-stage cyst cells (CC). The GB proliferates through exactly four rounds of TA-divisions and produces a cluster of 16 spermatogonia (SGs). After ceasing mitosis, the SGs enter terminal differentiation. They become spermatocytes (SCs), grow in size, undergo meiosis I and II, and finally differentiate into elongated spermatids. The surrounding cyst cells differentiate into late-stage cyst cells. (B) Apical region of a wildtype testis showing the germline cells in green (anti-Vasa) and the surrounding cyst cells in red (anti-GFP, UAS-GFP is driven by a cyst cell Gal4-transactivator, *C784*-Gal4). (C–F) Whole testes stained with DAPI. Genotypes and temperature regimens as indicated. Arrowheads: nuclei of early-stage germline cells, small arrows: spermatocyte nuclei, large arrows: spermatid heads, asterisks: apical tips of the testes. (C) A wildtype testis contains small, strongly DAPI-stained nuclei of early-stage germline cells exclusively in the apical region. (D) A *stet/stet*-testis is filled with small, strongly DAPI-stained nuclei. (E) A testis from a *stet/stet*; germline-sSpi-animal raised at 29°C appears wildtype. (F) A testis from a *stet/stet*; germline-sSpi animal raised at 29°C and shifted to 18°C with several clusters of small, strongly DAPI-stained nuclei. Scale bars: 30 µm.

Within the *Drosophila* testis, the germline cells and their somatic support cells are arranged in a spatio-temporal order along the apical to basal axis. The germline stem cells (GSCs) are attached to a single group of post-mitotic, apical hub cells and enclosed by cytoplasmic extensions from two somatic stem cells, the cyst stem cells (CySCs, Figure 1A) [9], [10]. Both stem cell populations undergo asymmetric mitotic cell divisions, producing gonialblasts and cyst cells respectively [11], [12]. Once produced, cyst cells normally cease mitosis and form the germline microenvironment. During this process, two cyst cells grow cytoplasmic extensions around one newly formed gonialblast [9], [13]–[15]. The cyst (composed of germline and two surrounding cyst cells) then undergoes a highly coordinated differentiation program. The cyst cells grow in size and continue to enclose the germline cells (Figures 1A, 1B) as they develop from early-stage cyst cells into late-stage cyst cells based on the size of their nuclei and the expression of stage specific molecular markers [8], [16], [17]. The enclosed gonialblast first proliferates by transit amplifying divisions (TA-divisions), which are a characteristic feature observed in most stem cell daughter populations. TA-divisions normally precede the second phase of tissue homeostasis, terminal differentiation, during which the cells undergo tissue-specific morphological changes to become specialized cells [2], [10], [18]–[21]. The correct transitions of cells from exiting the stem cell fate, through TA-divisions, and into terminal differentiation need to be tightly regulated to ensure the efficient production of specialized cells and to prevent tumorous growth of a tissue [22], [23]. A *Drosophila* gonialblast goes through exactly four rounds of synchronous TA-divisions with incomplete cytokinesis so that its progeny, the spermatogonia, remain interconnected by cytoplasmic bridges as they develop from 2-cell spermatogonia into 16-cell spermatogonia (Figure 1A). Spermatogonia are readily visible as small, round cells in the apical region of a wildtype testis (Figure 1B). After mitosis, the 16 interconnected spermatogonia enter terminal differentiation. The germline cells are now referred to as spermatocytes. Spermatocytes first grow in size and produce the majority of mRNAs and proteins required for the subsequent steps in differentiation. The spermatocytes are significantly larger cells than the spermatogonia and located further away from the apical tip than the spermatogonia (Figure 1B). After growth, the spermatocytes undergo the two divisions of meiosis and differentiate into elongated spermatids (Figure 1A) [9], [10]. Germline and cyst cells dissociate from each other only at the end of spermatogenesis for sperm individualization and release [8], [24], [25].

The cell fate decision of the GSC daughters to either self-renew or to initiate development depends on signals from the hub and the CySCs. The hub cells signal via the Signal Transducer and Activator of Transcription (JAK/STAT) and the Hedgehog signaling pathways to induce and maintain stem cell fate in the CySCs [26]–[30]. CySCs then signal for stem cell fate and maintenance to the enclosed GSCs via the Transforming Growth Factor β (TGFβ) signaling pathway [28], [31], [32]. Several lines of evidence suggest that the exit of the spermatogonia from TA-divisions is also regulated by TGFβ signaling [33]–[35]. However, in this case, the TGFβ pathway is activated in the surrounding cyst cells [36]. Thus, the literature provides some understanding of how communication between germline and soma regulates critical steps in tissue homeostasis, but the concepts governing how the cysts proceed from a precursor state (GSCs surrounded by CySCs), through the proliferative phase (spermatogonia surrounded by early-stage cyst cells) to differentiated cysts (spermatocytes surrounded by late-stage cyst cells) have not been fully addressed.

In both genders of *Drosophila*, germline differentiation is dependent on EGF signaling, a highly conserved pathway that plays multiple roles in development and has been associated with many forms of human cancers [14], [37]–[43]. The major ligand for the pathway, Spitz (Spi), and the EGFR are ubiquitously expressed in many tissues while pathway activation depends on the activity of cell-type specific ligand processing proteases [41], [44], [45]. In testes, Spi is activated by the germline-specific protease, Stet, and stimulates the EGFR on CySCs and cyst cells [14], [39], [40], [46], [47]. We previously showed that EGF signaling regulates cyst formation. In *spi* or *stet* mutant animals, germline cells and cyst cells were present but the cyst cells did not enclose the germline cells [14], [48].

It appears essential that exactly two cyst cells enclose the germline cells. The two cyst cells express different molecular markers and eventually become morphologically distinct, as one of them will develop into a relatively small head cyst cell and the other will develop into a much larger tail cyst cell [8], [17]. Temperature-sensitive mutations in the *egfr* and in *spi* led to defects in germline enclosure, as multiple cyst cells were associated with one cluster of germline cells. In both situations, the germline cells accumulated at early stages and failed to differentiate [39], [48]. A different effect on cyst cells was observed in animals mutant for *raf*, which encodes a Mitogen Activated Protein Kinase (MAPK) that can act downstream of the EGFR [49]. Testes from animals mutant for *raf* contained somatic cells in the CySC position that expressed late stage cyst cell markers. Though it was not addressed whether the cyst cells in *raf* mutant testes enclosed the germline cells, the testes contained over-proliferating germline cells similar to testes from *egfr* and *spi* mutant animals [40]. Together, these observations suggested that signals from the cyst cells to the germline cells are essential for restricting germline proliferation. However, the literature does not reveal if overproliferation of the germline cells in the *spi, egfr,* or *raf* mutant animals was due to defects in germline-soma association or due to a direct requirement for EGF signaling in the cyst cells for the production of these signals.

To separate the role of EGF signaling during germline enclosure from the potential role of EGF past the enclosure event, we specifically addressed the behavior of the germline cells when properly enclosed by cytoplasmic extensions from exactly two early-stage cyst cells, as normally seen in wildtype testes. We discovered that EGF signaling has a dose-dependent effect on cyst development. When we reduced EGF signaling in testes, the germline cells appeared to be properly enclosed by cyst cells but were trapped in TA-divisions. When we increased EGF signaling in testes, the germline cells also appeared properly enclosed by cyst cells but the cysts entered terminal differentiation before the germline cells completed all four rounds of TA-divisions. These observations strongly suggest that EGF signaling from the germline to the cyst cells normally increases as the cysts develop and that this increase in EGF signaling leads to the production of different return signals with different effects on the germline. We further observed that simultaneous over-expression of EGF and EGFR promoted germline differentiation, while high levels of either EGF or EGFR alone had no effect on germline development. We conclude that the doses of EGF signaling are controlled at the level of both the active ligand produced by the germline cells and the available receptor molecules on the cyst cells as these two cell lineages develop. We propose that the increase in EGF signaling provides a temporal signature that guides the cysts through the early steps of development. This is a novel concept that may shed light on the principles how stem cell daughters differentiate into highly specialized cells.

## Results

### EGF Signaling from the Germline to the Surrounding Cyst Cells is Required for Spermatogonia to Proceed through TA-divisions

To investigate a dose-dependent effect of EGF signaling we used the UAS/Gal4- system that allows for temporal control of tissue-specific expression of target genes by exposing *Drosophila* to different temperatures. When *Drosophila* are exposed to a temperature of 18°C, the Gal4 transcription factor has low activity. In contrast, when *Drosophila* are exposed to a temperature of 29°C, Gal4 is highly active [50]–[52]. To address whether and how EGF signaling plays a role in cyst development past the enclosure event, we first generated testes in which we decreased EGF signaling and investigated the resulting effect on the cysts.

In wildtype testes, early-stage germline cells have small, bright nuclei (Figure 1C, arrowhead) when stained with the DNA-dye 4, 6-diamidino-2-phenylindole (DAPI). The more basally located spermatocytes have larger, less bright DAPI-stained nuclei (Figure 1C, small arrow), and the bundles of spermatids at the base have sickle-shaped DAPI-stained nuclei (Figure 1C, large arrow). We previously established that testes from *stet^1^* mutant animals (*stet/stet*) are short and contain only cells with small, bright DAPI-stained nuclei (Figure 1D, arrowheads), while testes from *stet^1^* mutant animals expressing constructs for either the Stet protease or for secreted EGF ligand (*stet/stet;* germline*-*sSpi) are indistinguishable from wildtype testes when raised at 29°C (Figure 1E) [14]. We reasoned that Gal4 activity should decrease or cease upon shifting *stet/stet;* germline*-*sSpi-animals from 29°C to 18°C. The germline cells within one cyst in *stet/stet;* germline*-*sSpi-testes should continue to undergo TA-divisions but eventually have a lower dose of Spi compared to germline cells within a cyst in control testes. Hence a mutant phenotype should develop.

Testes from control siblings did not display any defects in spermatogenesis when shifted from 29°C to 18°C. However, after one day at 18°C, testes from *stet/stet;* germline*-*sSpi-animals frequently contained clusters of cells with small, bright DAPI-stained nuclei (Figure 1F, arrowheads, 80% of testes, n>100). We do not have the tools to measure the amount of Spi molecules. However, the appearance of the clusters of over-proliferating germline cells in *stet/stet;* germline*-*sSpi-testes shifted from 29°C to 18°C strongly suggests that the level of Spi in these testes is lower than the level of Spi molecules in a wildtype testes. Similar clusters of over-proliferating germline cells were observed in a variety of genetic backgrounds in which EGF signaling was reduced (Table 1), confirming that their appearance was not due to a genetic background mutation but characteristic for defects in EGF signaling.

**Table 1:** Summary of genotypes examined.

Genotype	Temperature Regimen
*stet ^1^, Nanos-Gal4*/*stet ^1^*, UAS-sSpi	Shifted from 29°C to 18°C
*stet ^1^/stet ^3^*	Raised at any temperature
*stet ^2^/stet ^3^*	Raised at any temperature
*spi ^77-20^/spi ^77-20^*	Raised at 21°C to 26.5°C
*Nanos*-Gal4-UAS-Spi-RNAi ^103817^	Shifted from 18°C to 26.5°C
*C784*-Gal4/UAS-EGFR-RNAi ^TRIP.JF02283^	Shifted from 18°C to 26.5°C
*C784*-Gal4/UAS-EGFR-RNAi ^TRIP.JF02284^	Shifted from 18°C to 26.5°C
*C784*-Gal4/UAS-Rolled-RNAi ^TRIP.HMS007^	Shifted from 18°C to 29°C

Listing of animal genotypes examined and the temperature regimen under which their germline cells were investigated. The majority of the genotypes were shifted to a different temperature as one to seven day old adults. Testes were dissected seven days after the temperature shift. *stet* or *spi* mutant animals without expression constructs were raised to adulthood and dissected within one to seven days after hatching. For each genotype, testes from at least 30 animals were examined. Though the severity of the phenotypes of the different genotypes varied, they all contained clusters of over-proliferating early-stage germline cells among otherwise wildtype appearing germline cells.

**Figure 2:**
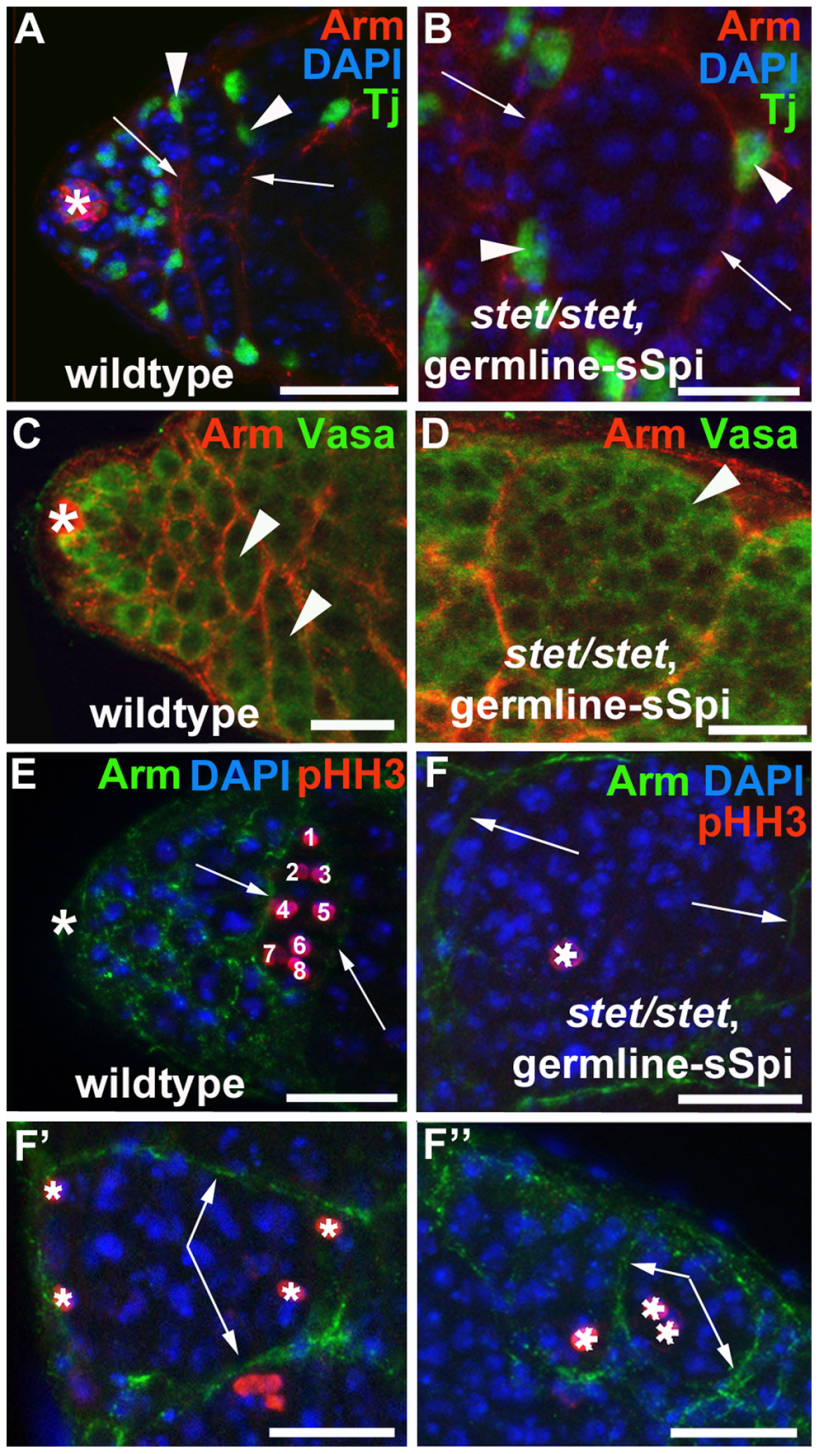
The germline cells within cysts in testes with decreased EGF signaling divided out of synchrony. (A–F′′) Germline and cyst cells in testes from wildtype animals and *stet*, germline-sSpi-animals shifted from 29°C to 18°C for two days. Genotypes and stainings as indicated. (A, B) In (A) the apical region of a wildtype testis and B) a *stet/stet*, germline-sSpi-testis, germline cells (blue) are enclosed by Arm-positive (red) cytoplasmic extensions (arrows) from two Tj-positive (green) cyst cells (arrowheads). Note that the cyst in the *stet/stet*, germline-sSpi-testis contains more than 16 early-stage germline cells. (C, D) The Arm-enclosed (red) germline cells (arrowheads) express the germline-specific marker Vasa (green) in testes from (C) wildtype and (D) *stet/stet*, germline-sSpi-animals. (E–F′′) In a (E) wildtype testis, all spermatogonia (blue) within a single cyst are in division (red, and numbered 1–8), while in (F-F′′) *stet/stet*, germline-sSpi-testes, only a fraction of the germline cells within one cyst divide (red and marked by asterisks). Note that the germline cells are enclosed in Arm-positive cyst cell cytoplasmic extensions (green). Large asterisks: apical tips of the testes, small arrows: cytoplasmic extensions of cyst cells, scale bars: 30 µm.

In wildtype testes and testes from *stet/stet;* germline*-*sSpi-animals raised at 29°C, we detected two cyst cells positive for the nuclear cyst cell marker Traffic jam (Tj, Figure 2A, arrowheads) associated with each cluster of transit amplifying spermatogonia (blue in Figure 2A) [53]. The two cyst cells enclosed the spermatogonia in cytoplasmic extensions, as visualized by the cell surface marker Armadillo (Arm, Figure 2A, arrows) [54]. Germline cells and cyst cells remained properly associated in *stet/stet;* germline*-*sSpi-testes even after shifting the animals to 18°C for one or two days. In these testes, the clusters of over-proliferating early stage germline cells (blue in Figure 2B) were still enclosed by cytoplasmic extensions from two cyst cells, based on anti-Tj (Figure 2B, arrowheads) and anti-Arm staining (Figure 2B, arrows). We conclude that shifting *stet/stet;* germline*-*sSpi-animals to 18°C does not disrupt germline enclosure, but does disrupt germline differentiation.

All cells within each enclosed cluster of over-proliferating germline cells in *stet/stet;* germline*-*sSpi-testes were small and expressed germline-specific markers, such as anti-Vasa (compare Figure 2D to 2C) [55]. The enclosed germline cells, however, did not undergo all four rounds of TA-divisions. In a wildtype testis, each GSC normally divides asynchronously to produce two distinct daughter cells, a new GSC and a gonialblast. The gonialblast and its daughters, in contrast, divide in a synchronous manner to produce equal precursor cells, the spermatogonia [10]. When control testes from wildtype animals were labeled with the M-phase marker anti-phosphorylated Histone H3 (pHH3), pHH3-positive spermatogonia were always seen in groups of two, four, or eight cells (Figure 2E, numbered 1–8, n>100). In testes from *stet/stet;* germline*-*sSpi-animals shifted from 29°C to 18°C for one or two days, only a fraction of the germline cells (Figure 2F–F′′, blue) within a single cyst were positive for pHH3 (Figure 2F–2F′′, pHH3-positive cells are marked by asterisks, n>20). We only detected either single pHH3-positive cells within a cyst containing over-proliferating germline cells (Figure 2F), several single pHH3-positive cells within a cyst containing over-proliferating germline cells (Figure 2F′), or small groups of pHH3-positive cells within a cyst containing over-proliferating germline cells (Figure 2F′′ shows an example of a cyst containing two pHH3-positive cells, next to a cyst with only one pHH3-positive cell). Confirming the role for EGF signaling in guiding TA-divisions, we observed the same phenotype in the testes of animals carrying a temperature sensitive allele of *spi, spi^77-20^* (*spi/spi*), raised at semi-permissive temperature (data not shown). These findings strongly suggest that EGF signaling within the cysts is required for the production of a return signal that allows the germline cells to undergo all four rounds of TA-divisions. They further suggest that the dose of EGF signaling required for the production of this return signal is higher than the dose of EGF signaling required for germline enclosure.

### A High Dose of EGF Signaling caused Spermatogonia to Bypass TA-divisions

To generate cysts with increased EGF signaling we also took advantage of the UAS-Gal4-system. Flies carrying constructs for the expression of constitutively active versions of the EGFR (caEGFR), UAS-EGFR^λtop^ and UAS-EGFR^A887T^, cause hyperactivity of the EGF pathway when expressed in *Drosophila* ovaries or eyes [56], [57]. We expressed these two well-established tools, as well as constructs expressing secreted Spi (UAS-sSpi) and a wildtype version of the EGFR (UAS-EGFR) in testes of otherwise wildtype animals. The constructs were specifically expressed in the cyst cells using two different cyst cell Gal4-transactivators, *C784*-Gal4 and *tj*-Gal4 (cyst cell*-*caEGFR-testes and cyst cell-sSpi/EGFR-testes). To circumvent expression of the constructs during development, animals were raised at 18°C and also carried a ubiquitously expressed, temperature sensitive Gal80 (*tub*-Gal80^ts^), a known negative regulator of Gal4, that has high activity at 18°C and low activity at 29°C [52], [58].

**Figure 3:**
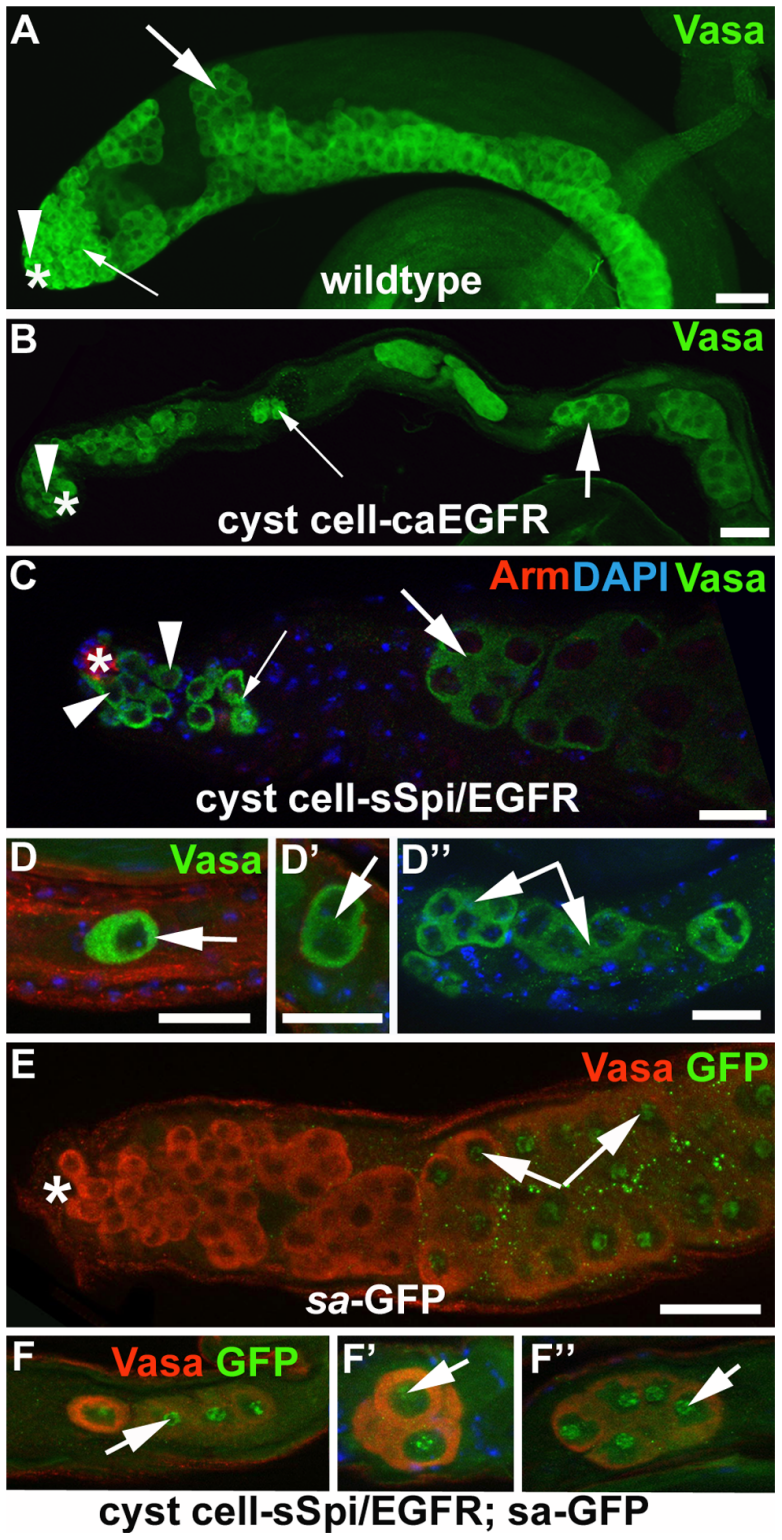
A high dose of EGF signaling promoted differentiation of germline cells into the spermatocyte stage. (A–C) Apical regions of testes showing Vasa-positive germline cells (green). Note that (B) cyst cell-caEGFR-testes and (C) cyst cell-sSpi/EGFR-testes raised at 18°C and shifted to 29°C for seven days contain fewer spermatogonia and spermatocytes compared to a (A) wildtype testis. (D–D′′) Germline cells along the testis coil are the size of spermatocytes and found in groups of less than 16 cells. (E) Spermatocytes but not the early germline cells in the apical region of a control testis express GFP (arrows) from a *sa*-GFP construct. (F–F′) The large germline cells along the coil of cyst cell-sSpi/EGFR-testes express GFP from the *sa*-GFP construct (arrows). Genotypes as indicated. Arrowheads: GSCs, small arrows: spermatogonia, large arrows: spermatocytes. Asterisks: apical tips of the testes, scale bars: 30 µm.

Testes from adult wildtype and sibling controls (non-shifted cyst cell*-*caEGFR-animals, non-shifted cyst cell-sSpi/EGFR-animals, animals carrying only the Gal4-transactivators or only the UAS-constructs shifted to 29°C for seven days) did not display observable defects in spermatogenesis. They contained Vasa-positive GSCs (Figure 3A, arrowhead), gonialblasts, and spermatogonia (Figure 3A, small arrow) in the apical region, followed by clusters of spermatocytes (Figure 3A, large arrow) in more basal regions of the testes. Testes from cyst cell-caEGFR-animals and from cyst cell-sSpi/EGFR-animals shifted to 29°C for seven days were much thinner than wildtype testes and contained fewer germline cells than control testes (Figures 3B, 3C). We detected single germline cells in the apical region of cyst cell-caEGFR-testes and cyst cell-sSpi/EGFR-testes (Figures 3B, 3C, arrowheads), followed by very few clusters of spermatogonia (Figures 3B, 3C, small arrows), and, more basally, clusters of spermatocytes (Figures 3B, 3C, large arrows, Table 2).

**Table 2:** Summary of over-expression of EGF signaling components and the observed effects on the germline cells.

Genotype	Few SGs	<16SCs/cyst
*C784*-Gal4/*tub*-Gal80 ^ts^; UAS-EGFR ^A887T^	Yes	Yes
*Tj*-Gal4/*tub*-Gal80 ^ts^; UAS-EGFR ^A887T^	Yes	Yes
*C784*-Gal4/*tub*-Gal80 ^ts^; UAS-EGFR ^λtop^	Yes	Yes
*Tj*-Gal4/*tub*-Gal80^ts^; UAS-EGFR ^λtop^	Yes	Yes
*C784*-Gal4/*tub*-Gal80 ^ts^; UAS-sSpi/UAS-EGFR	Yes	Yes
*Tj*-Gal4/*tub*-Gal80 ^ts^; UAS-sSpi/UAS-EGFR	Yes	Yes
*Nanos*-Gal4-UAS-sSpi/UAS-EGFR	No	No
*C784*-Gal4/*tub*-Gal80 ^ts^; UAS-EGFR	No	No
*Tj*-Gal4/tub-Gal80 ^ts^;UAS-EGFR	No	No
*C784*-Gal4/t*ub*-Gal80 ^ts^; UAS-EGFR/UAS-GalH	No	No
*C784*-Gal4/tub-Gal80 ^ts^;UAS-sSpi	No	No
*Tj*-Gal4/tub-Gal80 ^ts^;UAS-sSpi	No	No
*C784*-Gal4/tub-Gal80 ^ts^;UAS-sSpi/UAS-sSpi	No	No

Animals from various genotypes were shifted from 18°C to 29°C within one to seven days after hatching and their testes investigated at seven days after the shift. Only expression of caEGFR or co-expression of Spi and EGFR in the cyst cells produced mutant phenotypes (n>30). The testes contained fewer spermatogonia (SGs) compared to controls and contained clusters of less than 16 spermatocytes (SCs) per cyst.

Many of the spermatocytes in cyst cell-sSpi/EGFR-testes were single (Figure 3D, arrow), or in clusters of only two (Figure 3D′, arrow), four (Figure 3D′′, arrows), or eight cells instead of the normal 16, 32, or 64-cell clusters and expressed spermatocyte-specific molecular markers, such as a *spermatocyte arrest*-Green Fluorescent Protein (*sa*-GFP, compare Figures 3F–F′′ to Figure 3E, arrows point to GFP in the germline nucleoli) [59]. Our data suggest that a high dose of EGF signaling caused the germline cells to enter the spermatocyte differentiation program prior to completing all four rounds of TA-divisions. Single spermatocytes and small clusters of spermatocytes were detected in 95% of the cyst cell*-*sSpi/EGFR-testes (n = 76) but were not found in testes from *w^1118^*-animals (n = 80), in testes from cyst cell*-*sSpi/EGFR-animals prior to the temperature shift (n = 37), or in control testes from animals carrying only the Gal4-transactivators (n = 42) or only the UAS-constructs (n = 58) shifted to 29°C. Thus, their appearance in the testes of cyst cell*-*sSpi/EGFR-animals shifted to 29°C was specifically due to the increase in EGF signaling. Conversely, over-expression of sSpi and EGFR in the germline via the *nanos*-Gal4 transactivator did not result in any observable defects in the testes (Table 2). This confirms that the mutant phenotype was due to increased EGF signaling in cyst cells.

**Figure 4:**
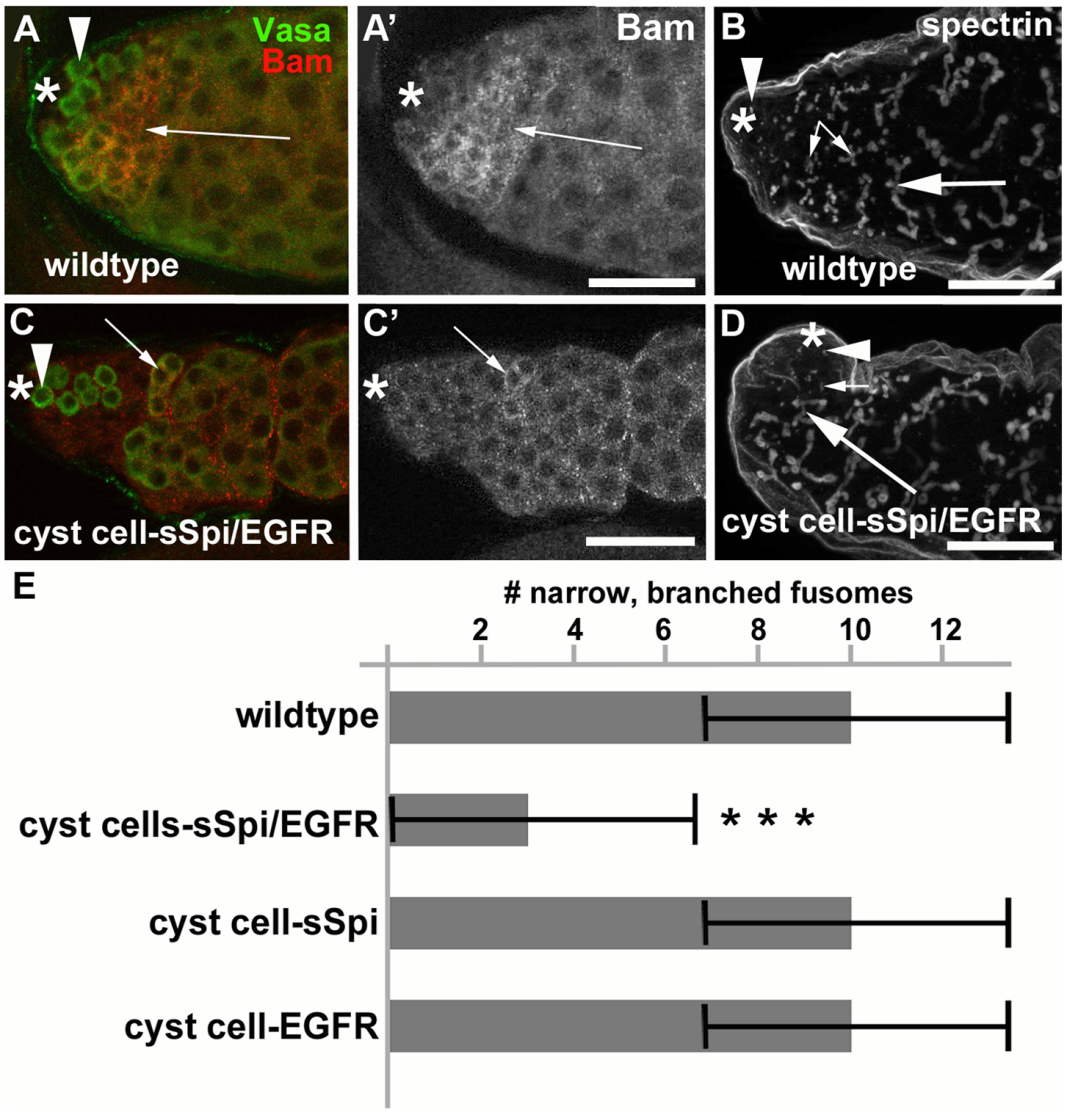
Testes with increased EGF signaling contained few spermatogonia. (A) A wildtype testis showing co-expression of Vasa (green) and Bam (red) in many germline cells in the apical region. (A′) Same testis as in (A) showing Bam expression only. B) A wildtype testis labeled with antibodies against α-spectrin. Arrowhead: GSC containing a round fusome, small arrows: narrow fusomes interconnecting spermatogonia, large arrow: wide fusomes interconnecting spermatocytes. (C) A cyst cell-sSpi/EGFR-testis showing co-expression of Vasa (green) and Bam (red) in only a few germline cells. (C′) Same testis as in (C) showing Bam expression only. (D) A cyst cell-sSpi/EGFR-testis showing the presence of only few narrow fusomes (arrows), and the presence of GSCs with a round fusome (arrowhead), and spermatocytes with wide fusomes (large arrow). (E) Bar graph showing the differences in numbers of narrow branched fusomes between testes from control and experimental animals. Three asterisks mark a statistically significant difference compared to the control. Genotypes and stainings as indicated, asterisks in (A-D): apical tips of the testes, scale bars: 30 µm.

Testes from cyst cell-sSpi/EGFR-animals already contained fewer germline cells in the apical region after only two days at 29°C, based on expression of the molecular germline markers Vasa, *bag-of-marbles* (*bam*), and α-spectrin. Wildtype testes contain a wide area of cells that co-express Vasa and the spermatogonial marker Bam in the apical region (Figures 4A, 4A′, arrow) [60], [61]. In addition, the spermatogonia contain α-spectrin-positive fusomes of characteristic shape and size [62]. In wildtype testes, the GSCs at the apical tip contained round α-spectrin-positive fusomes (Figure 4B, arrowhead), the spermatogonia contained narrow, branched α-spectrin-positive fusomes (Figure 4B, small arrows), and the spermatocytes contained wide, branched α-spectrin-positive fusomes (Figure 4B, large arrow). After only two days at 29°C, testes from cyst cell-sSpi/EGFR-animals contained very few cells in the apical region that co-expressed Vasa and Bam (Figures 4C, 4C′, arrow). Furthermore, these testes contained hardly any narrow α-spectrin-positive fusomes, normally found in spermatogonia (Figure 4D, small arrow). The cyst cell-sSpi/EGFR-testes did contain round α-spectrin-positive fusomes at the apical tip, indicating that GSCs were present (Figure 4D, arrowhead) and wide, branched α-spectrin-positive fusomes, as normally found in spermatocytes (Figure 4D, large arrow). To demonstrate the difference in the number of spermatogonia found in control and cyst cell-sSpi/EGFR-testes, we counted and compared the narrow fusomes detected in a single focal plane per testis. Within one focal plane, wildtype testes contained an average of eight (+/−three) narrow, branched fusomes (n>50, Figure 4E). Cyst cell-sSpi/EGFR-testes either lacked, or contained less than five fusomes (average of three (+/−three) within one focal plane (n = 36, Figure 4E). The difference in the number of narrow fusomes is statistically relevant, with a P-value <0.0001 (according to the student’s t-test, and marked by asterisks). Together, our data suggest that by two days after the temperature shift, many of the spermatogonia had already differentiated into spermatocytes. Consistent with the idea that the spermatogonia differentiated into spermatocytes, no apparent hallmarks of apoptotic cell death were observed in testes from cyst cell*-*sSpi/EGFR-animals (n = 50) compared to control-animals (n = 50) at one, two, or seven days after the shift to 29°C on the basis of the cell death assay, TUNEL (not shown).

### A High Dose of EGF Signaling Promoted Cyst Cell Differentiation

**Figure 5:**
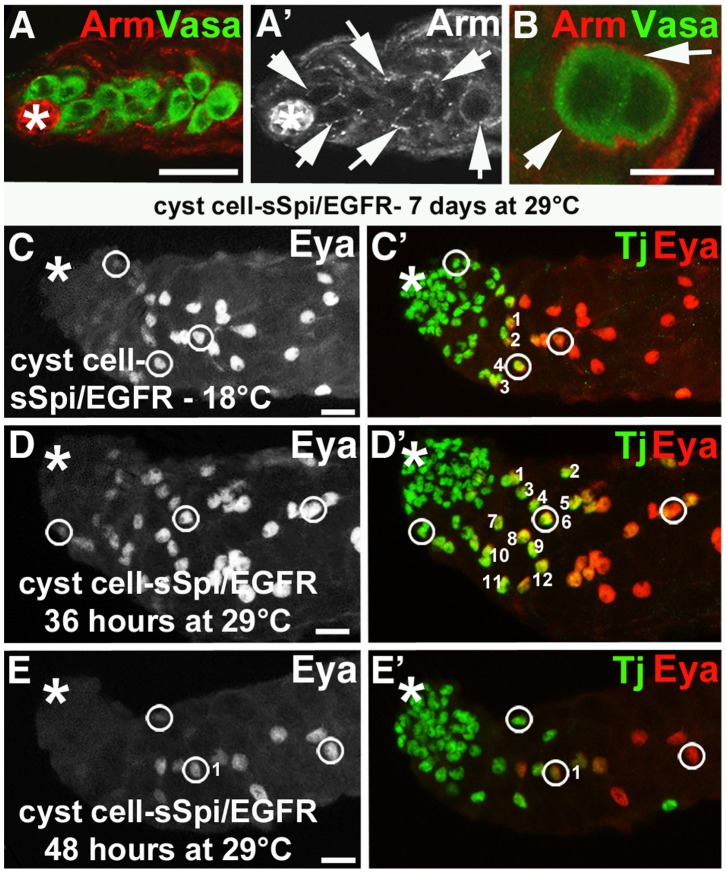
A high dose of EGF signaling promoted cyst cell differentiation. (A) Apical region of a testis from a cyst cell-sSpi/EGFR-animal showing that the germline cells (green) are enclosed by Arm-positive cytoplasmic extensions (red) of cyst cells. (A′) Same testis as in (A) showing the Arm-positive cytoplasmic extensions of cyst cells (arrows) only. (Note that not all cytoplasmic extensions are in focal plane) (B) A cluster of two spermatocytes (green) in a cyst cell-sSpi/EGFR-testes is enclosed in Arm-positive cyst cell cytoplasmic extensions (red, arrows). (C–E′) Apical regions of testes labeled with (left panel) only Eya and (right panel) co-labeled with Eya (red) and Tj (green). Cyst cells appear to accumulate Eya as they differentiate. The left-most circles in each testis show the most apical cyst cell nuclei that express low levels of Eya. These early-stage cyst cells also express high levels of Tj. The middle circles show cyst cells in the growth phase that express high levels of both, Eya and Tj. Small numbers indicate the numbers of the Eya^+^, Tj^+^-cells in one focal plane. The right-most circles show cyst cells after the growth phase expressing high levels of Eya, but low levels of Tj. Genotypes as indicated, scale bars: 30 µm.

Communication between germline cells and cyst cells did not appear to be disrupted in cyst cell-sSpi/EGFR-testes as the germline cells and the cyst cells remained properly associated. The early-stage germline cells at the apical tip (Figures 5A, 5A′) and the small clusters of spermatocytes (Figure 5B) along the testes coil were both enclosed by cyst cell cytoplasmic extensions (arrows in Figures 5A′, B), even after seven days at 29°C. The cyst cells in cyst cell-sSpi/EGFR-testes appeared to differentiate along with the enclosed germline cells upon the temperature shift. After the germline cells cease TA-divisions and become spermatocytes, both the nuclei of the germline cells and the nuclei of the surrounding cyst cells grow in size (in the following referred to as the growth phase) [10]. Cyst differentiation is characterized by changes in the expression of the transcription factors, Tj and Eya. Tj is considered an early-stage cyst cell marker due to its expression in CySCs, cyst cells prior to the growth phase, and cyst cells during the growth phase [53]. Eya is considered a late-stage cyst cell marker as it is expressed at high levels in cyst cells after the growth phase [63]. We detected a low level of Eya in the small size cyst cell nuclei shortly before growth phase begins. During the growth phase, the level of Eya expression increased as the cyst cell nuclei became larger. After the growth phase, the large cyst cell nuclei continued to express Eya at high levels (Figure 5C, 5C′, compare the size and staining intensity of the cyst cell nuclei within the white circles). When control testes from cyst cell-sSpi/EGFR-animals raised at 18°C (n = 20) were double-labeled with Anti-Tj and anti-Eya, a small fraction of the cyst cell nuclei that are associated with germline cells expressed both markers (yellow in Figure 5). By analyzing each testis in all focal planes, we detected an average of 14 (+/− eight) cyst cells that were both Tj-positive and Eya-positive (Tj^+^, Eya^+^-cyst cells, note that the testis in Figure 5C′ shows four Tj^+^, Eya^+^-cyst cells in one focal plane). Testes from cyst cell-sSpi/EGFR-animals shifted to 29°C also contained Tj^+^, Eya^+^-cyst cells yet their numbers changed dependent on how long the animals were at 29°C. At 36 hours after the temperature shift, the cyst cell-sSpi/EGFR-testes (n = 20) contained more Tj^+^, Eya^+^-cyst cells than the control testes (compare Figures 5D, 5D′ to 5C, 5C′). Cell counts revealed an average of 22 (+/−11) Tj^+^, Eya^+^-cyst cells (statistical difference in the number of Tj^+^, Eya^+^-cyst cells compared to non-shifted cyst cell-sSpi/EGFR-testes: P<0.0001, according to the student’s t-test). This shows that over-expression of sSpi and EGFR increased the number of cysts that have entered the growth phase. By 48 hours after the temperature shift cyst cell-sSpi/EGFR-testes (n = 20) contained fewer Tj^+^, Eya^+^-cyst cells (Figure 5E, E′) with an average of only eight (+/− eight). These are statistically fewer Tj^+^, Eya^+^-cyst cells compared to Tj^+^, Eya^+^-cyst cells in non-shifted testes (P<0.001). This suggests that after 48 hours at 29°C most of the cyst cells that were associated with germline cells have differentiated into Tj-negative, Eya-positive late-stage cyst cells. These observations suggest that a high dose of EGF either directly or indirectly promotes cyst cell differentiation.

### The Reduction in Spermatogonia was Dependent on Co-expression of Both EGF and EGFR

**Figure 6:**
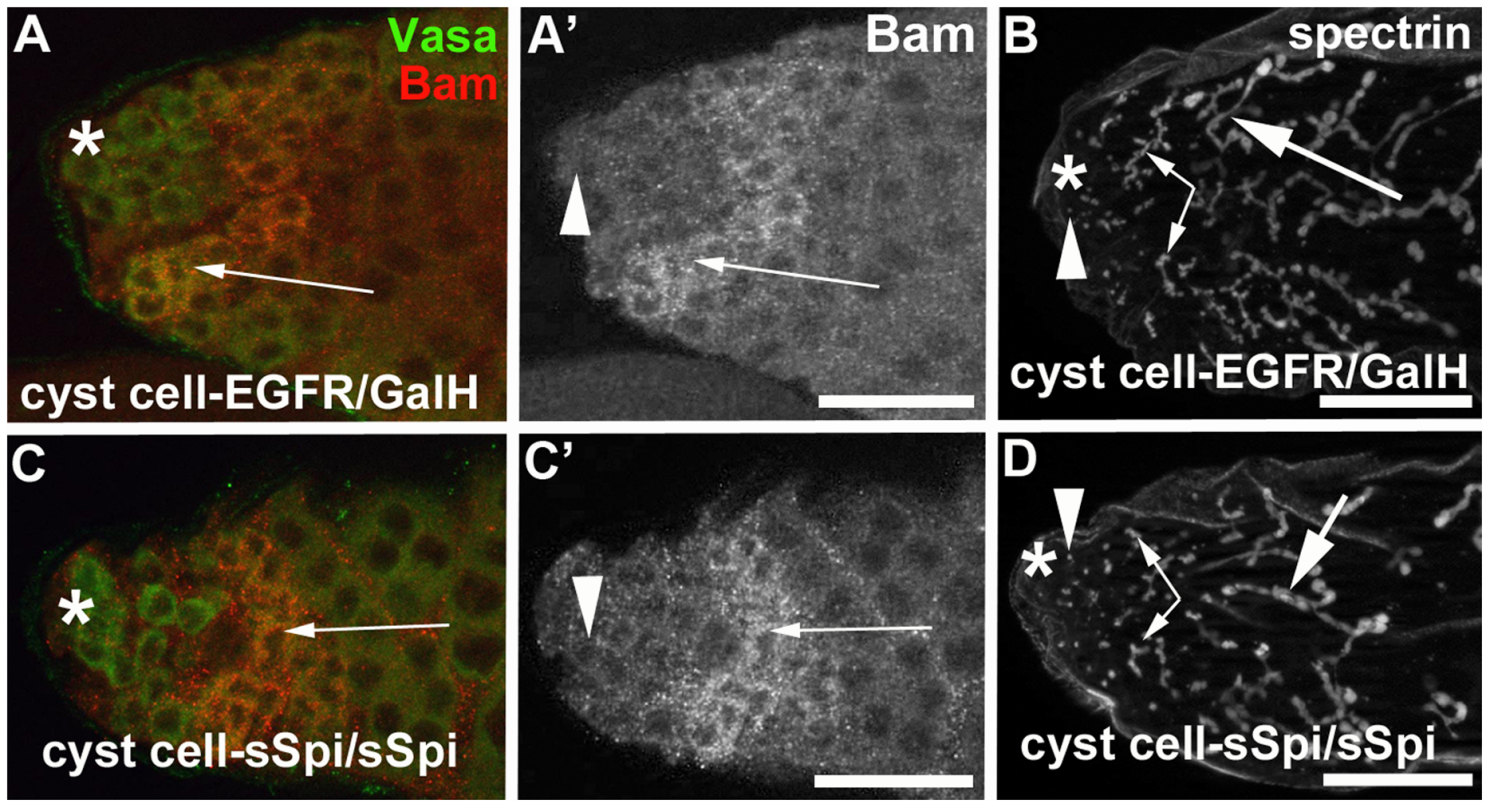
Increased EGF signaling depends on both the dose of EGF and the dose of EGFR. (A–D) In testes expressing either (A, A′, B) EGFR and GalH or (C, C′, D) two copies of sSpi, many Vasa-positive germline cells (green in A and C) also expressed Bam (A, A′, C, C′ arrows) and contained many narrow, branched α-spectrin-positive fusomes (B, D, small arrows). Arrowheads: round fusomes in GSCs, small arrows: narrow fusomes interconnecting spermatogonia, large arrows: wide fusomes interconnecting spermatocytes, asterisks: apical tips of testes, scale bars: 30 µm.

Interestingly, while the expression of caEGFR or the co-expression of sSpi and EGFR in cyst cells caused a phenotype shortly after the shift to 29°C, expression of sSpi (cyst cell*-*sSpi-testes) or the EGFR (cyst cell-EGFR-testes) alone using either of the two cyst cell Gal4-transactivators did not have an effect on the cysts (n>30, Table 2). For example, over-expression of only the sSpi-ligand or the EGFR in cyst cells did not cause a reduction in the number of narrow fusomes that interconnect spermatogonia. Testes from either genotype contained an average of eight (+/−three) thin, branched fusomes (n>30) in a single focal plane (Figure 4E) compared to controls with an average of eight (+/−three, Figure 4E). This is a surprising observation, as we should have the same number of Gal4 molecules in all experiments and the same or even higher levels of sSpi or EGFR expression as we used the same UAS-sSpi and UAS-EGFR insertions for these experiments. We next increased the amount of EGFR molecules produced in the cyst cells by co-expressing EGFR with GalH, a potentiator of Gal4 (cyst cell-EGFR/GalH) [64]. We did not observe an effect on the germline cells in testes from cyst cell-EGFR/GalH-animals (Table 2). Similarly, increasing the number of sSpi molecules by expressing two copies of secreted Spi in the cyst cells (cyst cell-sSpi/sSpi) also did not have an effect on the germline cells (Table 2). As in the controls, cyst cell-EGFR/GalH-testes (Figures 6A, 6A′, 6B) and cyst cell-sSpi/sSpi-testes (Figures 6C, 6C′, 6D) contained a wide area of spermatogonia co-expressing Vasa and Bam (arrows in Figures 6A, 6A′, 6C, 6C′) and contained many narrow fusomes as normally seen in interconnected spermatogonia (Figures 6B, 6D, small arrows). We conclude that the reduction in the number of early-stage cysts containing spermatogonia and the presence of late-stage cysts containing less than 16 spermatocytes was dependent on the over-expression of both the ligand and the receptor.

## Discussion

Here we propose that a temporal increase in EGF signaling between germline cells and surrounding cyst cells regulates cyst development. We present genetic evidence that a low dose of EGF signaling is required for the early stages of germline development, the progression of the germline cells through TA-divisions, and that a high dose of EGF signaling promotes the entry of germline and cyst cells into terminal differentiation. Reduction in EGF signaling led to the accumulation of cyst cell-enclosed, tumor-like aggregates of early-stage germline cells. The germline proliferation defects in EGF mutant testes were different from the germline proliferation defects reported for other mutants displaying germline tumors. For example, germline cells mutant for *bam* or *benign gonial cell neoplasm (bgcn)* over-proliferated beyond the 16-cell stage, producing large clusters of interconnected spermatogonia [60], [65]. The same phenotype was observed when germline cells were associated with cyst cells mutant for TGFβ signaling or when the germline cells overexpressed the TGFβ ligand *decapentaplegic* (*dpp*) [33], [36], [66]. Notably, the germline cells in testes with *dpp* over-expression divided in synchrony, as large cysts were detected in which all of the spermatogonia expressed the mitosis marker pHH3 [34]. In testes from *stet/stet;* germline*-*sSpi-animals shifted from 29°C to 18°C and in testes from *spi^77-20^*- animals raised at semi-permissive temperature, large cysts were detected as well. However, the germline cells within a single cyst divided out of synchrony as we observed that only a few of them were positive for pHH3. It has not been revealed yet how the decision of the gonialblast to divide synchronously is regulated. Synchronous versus asynchronous cell division could depend on morphology changes of the fusome. When a GSC or a gonialblast divides, the fusome morphology changes from spherical to dumbbell-shaped. During the division of a GSC, the fusome breaks and one spherical fusome is formed in each of the resulting daughter cells. When a gonialblast divides, the dumbbell structure of the fusome is maintained in the resulting 2-cell stage spermatogonia [62], [67]. We did not find indications that the asynchronous divisions observed in testes with reduced EGF signaling is due to a direct role of EGF in preventing fusome breakage in the germline cells. More likely than having a direct effect on fusome morphology, we hypothesize that EGF signaling regulates a return signal from the cyst cells to the germline cells that promotes synchronous cell divisions. We propose that EGF signaling normally regulates a return mechanism from the cyst cells that reduces stem cell characteristics in the spermatogonia which, in turn, promotes the progression of the spermatogonia through TA-divisions.

Conversely, a high dose of EGF signaling may decrease stem cell characteristics of the spermatogonia. We observed that germline cells in cyst cell-caEGFR-testes and in cyst cell-sSpi/EGFR-testes bypassed TA-divisions and developed into spermatocytes prior to the 16-cell stage. A similar effect on the germline cells was previously described for two other genetic backgrounds, the over-expression of *bam* and the loss of *nucleoporin98-96* (*nup98-96*). While loss of *bam* caused the proliferation of spermatogonia beyond the 16-cell stage, over-expression of Bam in the germline cells caused the differentiation of spermatogonia into spermatocytes at the 8-cell stage. This suggested that the germline-intrinsic levels of Bam regulate the number of TA-divisions [68]. In testes from animals mutant for *nup98-96*, the germline cells differentiated into spermatocytes at the two-, four-, and eight-cell stage instead of the 16-cell stage. This indicated that *nup98-96* plays a role in preventing the transcription or transport of differentiation factors into the nuclei of the spermatogonia [69]. The phenotypic similarity between testes from *bam* and *nup98-96* mutant animals and cyst cell-sSpi/EGFR-animals suggests that a mechanism exists that prevents the entry of spermatogonia into the spermatocyte-stage prior to the completion of exactly four rounds of TA-divisions and that the EGF pathway plays a role in this mechanism. However, how EGF signaling from the germline to the cyst cells ties into germline-intrinsic activities of Bam and Nup98-96 is yet to be explored.

**Figure 7:**
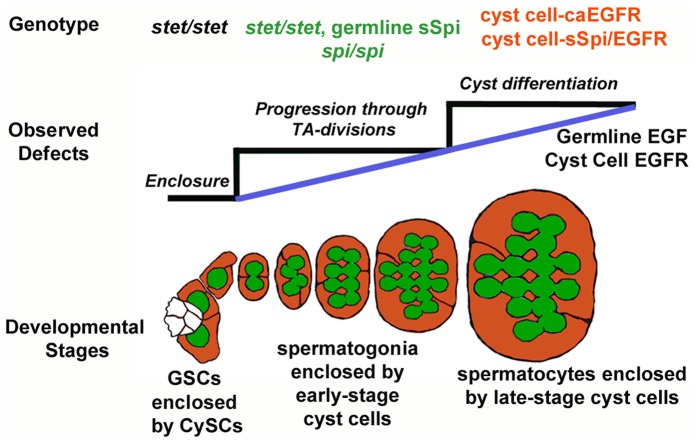
A temporal signature of EGF regulates early stages of cyst development. Model demonstrating the dose-dependent effects of EGF signaling on the germline and cyst cells as they develop through the early stages of spermatogenesis (bottom). Blue line indicates a gradual increase in EGF and EGFR, and black lines indicate increasing threshold doses of EGF and EGFR. Examples of genotypes in which the observed defects were studied are listed on top.

Together, our phenotypic analyses suggest that EGF signaling plays a rather complex role in the developing cysts. Based on the mutant phenotypes caused by the loss, reduction, and increase of EGF signaling, we propose that EGF signaling between germline and cyst cells increases as the cysts develop and that different doses of EGF signaling, in turn, induce distinct responses in the cyst cells: germline enclosure, the production of return signals that allow for the germline cells to progress through the early steps of development, the spermatogonia TA-divisions, and the production of return signals that promote terminal differentiation of the cysts (Figure 7). It is possible that the three different responses depend on three threshold levels of EGF signaling (black lines in Figure 7). Alternatively, a continuous increase in EGF signaling (blue line in Figure 7) may promote a continuous progression of the cysts towards terminal differentiation, thereby gradually reducing stem cell characteristics and increasing differentiation competence. The view that spermatogonia may retain stem cell characteristics is consistent with the observation that they can revert into GSCs when exposed to Unpaired, the ligand activating the JAK/STAT pathway [70]–[72].

Different doses of EGF signaling also guide the development of other tissues, for example photoreceptor development in the developing eye [57], [73]. Our findings suggest a different mechanism by which the different doses of EGF signaling are generated. In other *Drosophila* tissues, a spatial EGF gradient is generated by the diffusion of the ligand away from the source [73]–[75]. In testes, over-expression of either the ligand or the receptor alone was not sufficient to promote germline differentiation, arguing against a diffusion model. This is not surprising as the germline cells are completely enclosed by cyst cells and EGF thus cannot diffuse outside the cysts. Only when both the ligand and the receptor were overexpressed was a strong phenotype observed. We propose that different doses of EGF signaling are created by the coordinated increase of EGF signaling components in the germline cells and the cyst cells as they mature. This, in turn, could create a temporal signature that determines the behavior of the individual cysts. It remains to be ascertained if expression data, once reliable tools for imaging EGF expression levels or receptor stimulation become available, can confirm our hypothesis.

How could such an increase in ligand and receptor be regulated? A simple explanation for an increase in EGF would be that its amount depends on the number of germline cells. Within one cyst, the number of spermatogonia that can produce the active ligand increases during TA-divisions. At the 16-cell stage, the amount of ligand may be sufficient to induce the production of a signal in the cyst cells that promotes spermatocyte differentiation. However, such a simple mechanism is not likely as cysts in testes with reduced EGF signaling contained a large number of germline cells that eventually should have produced a high enough dose of EGF for differentiation, yet these germline cells still over-proliferated. More likely, the amount of EGF may depend either on transcriptional up-regulation of signaling molecules within the developing germline cells or on an increase of ligand secretion from the developing germline cells. Ligand secretion is a critical step during development of other *Drosophila* tissues, such as the eye and the nervous system. In the eye, for example, the cleaved Spi ligand is retained in the endoplasmatic reticulum to regulate the range of EGF signaling [76], [77]. In a similar manner, transcriptional up-regulation of the EGFR or a reduction in the amount of negative regulators of the EGFR may explain the increase in EGFR availability as the cysts develop. Along with this, the increase in the size of the cyst cells as they develop may serve as a vehicle for a more efficient display of EGFR molecules.

In summary, our data strongly support the idea that EGF signaling between germline cells and cyst cells increases as they transition from early to later stages of development, and that this increase guides early steps of gametogenesis. It will be interesting to learn if threshold doses of signaling molecules guide differentiation of stem cell lineages in mammals as well.

## Materials and Methods

### Fly Strains

Flies were raised on standard cornmeal molasses agar medium. *white^1118^*, *tub*-Gal80^ts^, UAS-Rolled-RNAi^TRIP.HMS007^, and balancer chromosomes are as described in [78] and were obtained from the Bloomington stock center. Likewise, UAS-EGFR [79], UAS-GalH [64], *C784*-Gal4 [80], and *nanos*-Gal4 [81] were obtained from the Bloomington stock center. UAS-EGFR-RNAi^TRIP.JF02283^ and UAS-EGFR-RNAi^TRIP.JF02284^ were obtained from the Harvard *Drosophila* RNAi Screening Center, UAS-Spi-RNAi^103817^ from the Vienna *Drosophila* Research Center, *tj*-Gal4 from the Kyoto Stock Center, UAS-*EGFR^A887T^* [57] from Nick Baker, and UAS-EGFR^λtop^ [56] from Trudi Schupbach. *stet-*alleles are described in [14] and the temperature sensitive *spi^77-20^*-allele is described in [48] **.** The UAS-sSpi-construct [82] was obtained from Ben Shilo and injected into flies by the company The Best Gene.

### UAS/Gal4 Expression Studies

Crosses were set up either at 18°C or at 29°C and the animals were shifted to different temperatures as outlined in the results.

### Immunofluorescence

Immunofluorescence experiments were performed as previously described [14]. Staining was observed with a Zeiss Axiophot microscope and images were taken with a CCD camera using an Apotome and Axiovision Rel Software. Single slices and z-stack-composites of up to ten slices (for DAPI and anti-α-Spectrin images) are shown. Mouse anti-α-Spectrin 3A9 (1∶10) developed by D. Branton and R. Dubreuil, mouse-anti-Bam (1∶5) developed by D. McKearin, and mouse anti-Armadillo (1∶10) developed by E. Wieschaus were obtained from the Developmental Studies Hybridoma Bank developed under the auspices of the NICHD and maintained by The University of Iowa, Department of Biological Sciences, Iowa City, IA 52242. Rabbit anti-phosphorylated Histone-H3 (Ser-10, 1∶500) was obtained from Millipore and goat anti-Vasa (1∶100) was obtained from Santa Cruz Biotechnology (dc-13). Guinea pig-anti-Tj (1∶5000) was a gift from Dorothea Godt. Fluorophore-coupled secondary antibodies (Molecular Probes) were used at 1∶1000.

### TUNEL-assay

Testes were dissected in Tissue Isolation Buffer [14], incubated with the TUNEL solutions following the manufacturer’s (Roche-Applied Scientific) instructions, and then used for immunofluorescence as described above.
